# Modelling the impact of protein-kinase R allelic variant on HIV biomarkers trajectories by means of latent class mixed models

**DOI:** 10.1038/s41598-022-23289-4

**Published:** 2022-11-03

**Authors:** Chiara Brombin, Sabrina Bagaglio, Federica Cugnata, Antonella Castagna, Caterina Uberti-Foppa, Stefania Salpietro, Clelia Di Serio, Giulia Morsica

**Affiliations:** 1 University Centre for Statistics in the Biomedical Sciences (CUSSB), Milan, Italy; 2 Vita-Salute San Raffaele University, Milan, Italy; 3 Department of Infectious Diseases, IRCCS San Raffaele Scientific Institute, Milan, Italy; 4 Biomedical Faculty, Università della Svizzera Italiana, 6900 Lugano, Switzerland

**Keywords:** Biomarkers, Statistics, Genetics, Risk factors, Infectious diseases

## Abstract

This paper is based on a retrospective longitudinal study on people living with HIV under antiretroviral treatment (ART) where allelic variants (either heterozygous CT genotype or homozygous CC genotype) have been evaluated at position −168 of the promoter region of the protein kinase R (−168/PKR). In general, antiviral effects of interferon are partially mediated by a RNA-dependent protein kinase (PKR) that, once activated, inhibits protein synthesis. Indeed, activation of PKR response can inhibit HIV replication. To explore the role of allelic variants in shaping dynamics of commonly monitored HIV biomarkers, CD4 cells, CD8 cells and HIV-load were modelled within a latent class mixed model (LCMM) to account for participants’ heterogeneity over time. The estimated models identified two sub-groups from CD4 and HIV-load dynamics, revealing better outcomes for subgroups of participants with the heterozygous CT genotype. Heterozygous CT subjects in one of the two identified subgroups exhibited higher increase of CD4 cells and more marked decrease of HIV-load, over time, with respect to the homozygous CC subjects assigned to the same group.

## Introduction

HIV infection and the progression to acquired immune deficiency syndrome (AIDS) have been the focus of deep genetic investigation. Although AIDS is not generally considered a genetic disease, its considerable heterogeneity is partially determined by gene variants that mediate virus replication and modulate immune response^1,2^.

HIV infected individuals progress to AIDS-defining disease after a variable time-interval. Rapid progressors develop AIDS in 1–5 years, whereas long-term ‘non-progressors’ may avoid AIDS for up to 20 years in absence of any ART^3,4^. Similarly, there are approximately 1% of people living with HIV (PLWH) who maintain undetectable HIV viral load (generally less than 50 copies/mL) for a prolonged period and are identified as elite controllers^5^.

On this ground, genome-wide association studies have been carried out to decipher host factors involved in the natural protection against disease progression.

The strength of the innate humoral and cell-mediated immune responses of infected individuals varies, as does the way in which they respond to anti-retroviral treatment.

The investigated candidate genes include MHC class I and class II genes, which have been shown to affect HIV susceptibility and disease progression because of peculiar combinations of “good” and “bad” alleles that determine the disease phenotype^6^. Other AIDS restriction genes (ARGs) have been identified. CC-type chemokine receptor 5 (CCR5) delta32 is a naturally occurring 32 bp knockout deletion variant: homozygotes resistant to R5-HIV-1 infection (the principal infecting HIV-1 strain) lack the requisite HIV-1 entry co-receptor CCR5 on their lymphoid cells^7,8^, whereas heterozygotes express less than half the wild-type levels of CCR5 receptors, thus slowing HIV-1 replication, spread and pathogenesis^9,10^.

The interferon system is a crucial component of the innate immune response to infectious agents. Interferon-induced, double-stranded, RNA-dependent protein kinase (PKR), the most widely studied member of the eIF-2alpha specific kinase subfamily^11,12^, whose gene is located on the short arm of chromosome 2, is a c-AMP independent, serine-threonine kinase characterised by two distinct kinase activities: autophosphorylation (which represents the activation reaction), followed by the phosphorylation of eIF2-alpha^13^, which can lead to limitations in functional eIF2-alpha and the inhibition of protein synthesis^14^. Protein kinase R may be activated during virus infection and thus plays a pivotal role in the regulation of protein synthesis in infected cells^15^.

A previous study^16^ when exploring the effect of interferon mediated mechanism of replication revealed that heterozygotes at position −168 in the promoter region of the PKR gene relative to the transcription initiation site were more likely to have a self-limited hepatitis C infection.

Since HIV and HCV share similar mechanisms of PKR inhibition, we investigated the distribution of allelic variants at position −168 within the promoter region of protein kinase R (−168/PKR). Their impact on longitudinal progression of HIV biomarkers is evaluated within a novel statistical procedure: Latent Class Mixed Models (LCMMs^17^). This modelling approach results to be powerful and flexible in handling data with longitudinal measurements allowing also for non-linear outcomes while uncovering latent homogeneous subgroups from a larger heterogeneous population. Indeed, the main output of the model is a data-driven procedure to select the optimal number of latent classes directly from the longitudinal course. LCMMs, differently from most statistical approaches, allow to gain information not just from observed covariates but directly from trajectories on different heterogeneous profiles. One main advantage of this procedure is that this approach allows to identify factors affecting the dynamics HIV biomarkers while exploring whether latent subgroups of participants with specific biomarkers dynamic exist.

## Results

### Study participants description

The characteristics at study entry of 92 Caucasian PLWH, on ART or commencing ART, are summarized in Table 1.

**Table 1:** Baseline characteristics of HIV-carriers.

Sex	Female	22 (23.9)
	Male	70 (76.1)
CCR5-delta32	*WT/delta32*	9 (9.8)
	*WT/WT*	83 (90.2)
Risk factor for HIV-1 infection	IVDU	44 (47.8)
	Sexual contact	48 (52.2)
anti-HCV	Neg	39 (42.4)
	Pos	53 (57.6)
HBsAg	Neg	88 (95.7)
	Pos	4 (4.3)
AIDS defining disease	No	67 (72.8)
	Yes	25 (27.2)
−168/PKR promoter gene	Homozygous CC	29 (31.5)
	Heterozygous CT	63 (68.5)
Age, years		43.0 [40–47]
Duration of HIV infection, years		14.08 [10.50–19.13]
CD4 cells count, cells/mm^3^		466 [311–657]
CD8 cells count, cells/mm^3^		1009 [752–1356]
CD4/CD8		0.49 [0.29–0.73]
HIV-RNA, log_10_ copies/mL		1.69 [1.69–2.57]
CD4 nadir cells count, cells/mm^3^		155 [67–277]
HIV-RNA, copies/mL	< 50	59 (64.1)
	≥ 50	33 (35.9)

Median and interquartile range [IQR] have been used for quantitative variables, while categorical variables have been summarized as frequencies and percentages (%).

*IVDU* intra venous drug users, *anti-HCV* hepatitis C virus antibodies, *HBsAg* hepatitis B surface antigen, *AIDS* acquired immune deficiency syndrome, *PKR* protein kinase R.

Study participants were prevalently males and had a relatively preserved immune status assessed by CD4 cells count and CD4/CD8 ratio. Most of participants at study entry had HIV-RNA < 50 copies/mL.

In particular, at study entry, the median HIV-RNA load was < 50 copies/mL and the third quartile < 400 copies/mL (see Table 1): this latter was considered an acceptable value in year 2005–2008, because ART was less effective before the introduction of integrase strand transfer inhibitors (INSTI). Additionally, 4/92 PLWH initiated ART at study entry. Median CD4 nadir was < 200 cells/mm^3^ with AIDS defining clinical condition in 27.2% of them.

With regard to treatment regimen at study entry: 39.1% of participants were on 1 or 2 antiretroviral drugs and 60.9% on at least 3 antiretroviral drugs (from the classes nucleos(t)ides reverse transcriptase inhibitors, NRTI; non-nucleoside reverse transcriptase inhibitors, NNRTI, protease inhibitors PI, fusion inhibitors FI, entry inhibitors EI, INSTI). This latter class of drugs was administrated in our centre starting from year 2008.

During a median follow-up (FU) of 13.17 years (IQR: 8.55–13.65) the median number of observations for CD4 cells count (total valid observations: 2970) was 35 (IQR: 24–42), for CD8 cells count (total valid observations: 2585) was 29 (IQR:19–37), for HIV-1 load (total valid observations: 2886) was 35 (IQR 22–41). During the period of observation 33 of 92 participants had at least one virological failure (VF). In 21/33 participants the first VF was consequent to low adherence to treatment; therefore, adherence was reinforced without changing ART regimen. Antiretroviral therapy was changed in the other 12 PLWH with VF during the FU (in 2 of these 12 cases ART change was due to drug toxicity).

Regarding the distribution of PKR genotype, 31.5% of participants were homozygotes CC and 68.5% heterozygotes CT. For CCR5-delta32 genotypes, the variant *WT/delta-32* was revealed in 9.8% of participants and the *WT/WT* in 90.2% of them.

### Effect of clinical characteristics and −168/PKR allelic variants on the dynamic of immune status and HIV-load

We fitted models for CD4, CD8 and HIV load entering the same set of covariates in all the models and specifying an increasing number of latent classes, from 1 to 3.

#### CD4 cell count dynamic and −168/PKR allelic variants

**Figure 1:**
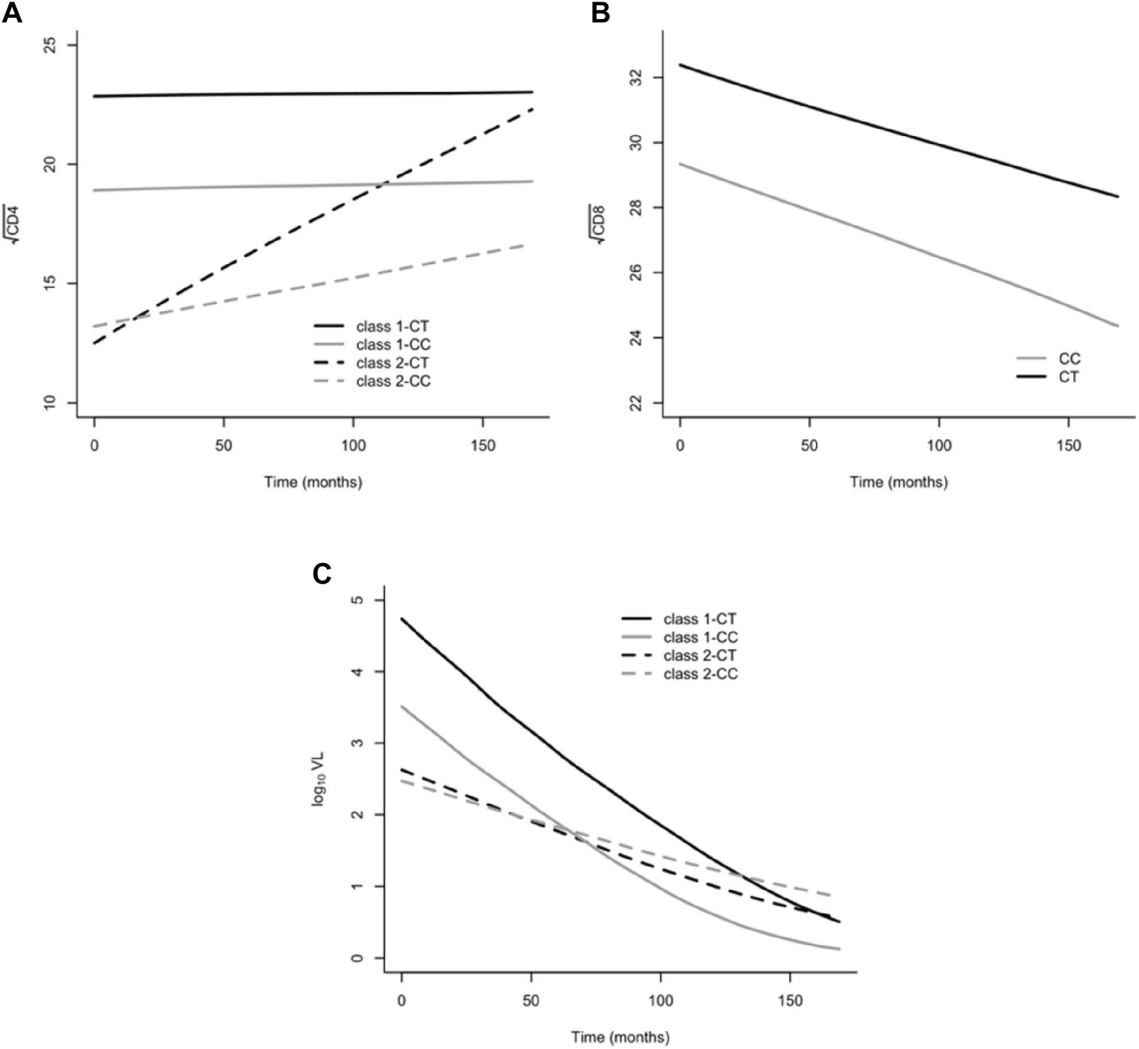
Class-specific model predicted curves for CD4 (**A**), CD8 (**B**) and HIV load (**C**) highlighting the effect of PKR genotype over time. To visualize the results, being in a multiple regression framework, the other model covariates were kept constant (age was set equal to 43 years, sex was fixed to male, disease duration from HIV diagnosis to study entry was set equal to 14 years, coinfection with hepatitis C and AIDS diagnosis were assumed to be present, the level homozygous for the delta 32 allele of CCR5 has been selected and sexual contact was set as route of infection). It should be noticed that since all these covariates are entered as fixed effects in the model, changes in their values have the only effect of shifting up/down the curves but curves shapes will looks exactly as shown below.

With regard to CD4 cells count, a two subgroups structure resulted to better capture the dynamic of this biomarker of HIV progression over time: 24 participants (26.09%) were assigned to class 1 and 68 (73.91%) to class 2 (see Table S1). Model of predicted class specific CD4 dynamic is reported in Fig. 1. Average posterior probabilities of falling into the class in which the subjects were classified are equal to 0.8751 and 0.9203, thus indicating an unequivocal classification.

We found that HIV transmission through a sexual contact (p = 0.0015), coinfection with HCV (p = 0.0147), diagnosis of AIDS (p = 0.0208) and age (p = 0.0076) were negatively associated with CD4 cells count, longitudinally evaluated. The CCR5/delta 32 genotype, instead, did not play a significant role in modulating CD4 cells count (Table 2). Although CD4 cells count showed an increasing trend over time, a significant increase of CD4 cells count was observed in participants with heterozygosity CT assigned to class 2 (p = 0.0081) but not in those assigned to class 1 subgroup (Table 2).

**Table 2:** Estimated latent class mixed model for CD4 (square root transformed) with two latent classes.

Parameter	Estimate	SE	p-value
intercept class1 (not estimated)	–		
intercept class2	− 1.8316	0.9088	0.0439
Sex (male vs. female)	− 0.2638	0.4394	0.5483
CCR5-delta32 polymorphism (*WT/WT* vs. *WT/delta32*)	− 0.5101	0.5610	0.3633
Risk factor for HIV infection (sexual vs. IVDU)	− 1.8592	0.5866	**0.0015**
Anti-HCV (pos vs. neg)	− 1.3182	0.5401	**0.0147**
AIDS defining disease (yes vs. no)	− 0.9438	0.4083	**0.0208**
−168/PKR genotype (CT vs. CC)-class1	1.4269	0.7923	0.0717
−168/PKR genotype (CT vs. CC)-class2	− 0.2102	0.4510	0.6412
Age, years	− 0.0764	0.0286	**0.0076**
Duration of HIV infection	− 0.0326	0.0379	0.3889
Time-class1	0.0016	0.0081	0.8491
Time-class2	0.0068	0.0043	0.1154
Time: −168/PKR genotype (CT vs. CC)-class1	− 0.0007	0.0090	0.9400
Time: −168/PKR genotype (CT vs. CC)-class2	0.0131	0.0050	**0.0081**

The “time: −168/PKR genotype” indicates the parameter for the interaction term, i.e., it describes the progression of CD4 separately for each allelic variant and for each latent class (class1 or class2).

*WT/WT* wild type, *anti-HCV* hepatitis C virus antibodies, *AIDS* acquired immune deficiency syndrome, *PKR* protein kinase R.

Significant coefficients are in bold.

#### CD8 cell count dynamic and −168/PKR allelic variants

When considering CD8 cells count (square root transformation has been applied), a one latent classes model was selected as the best to capture CD8 dynamic over time (see Table S1). A decreasing trend of CD8 cells count was observed over time (p = 0.0073, see Table 3). However, overall, on average, participants with the CT variant showed a higher CD8 cells count respect to those with the CC variant (p = 0.0378).

**Table 3:** Estimated latent class mixed model for CD8 (square root transformed) with one latent class.

Parameter	Estimate	SE	p-value
intercept (not estimated)	–		
Sex (male vs. female)	0.4939	0.4250	0.2452
CCR5-delta32 polymorphism (*WT/WT* vs*. WT/delta* 32)	0.1021	0.5303	0.8474
Risk factor for HIV infection (sexual vs. IVDU)	− 0.3013	0.5426	0.5787
Anti-HCV (pos vs. neg)	− 0.6821	0.5168	0.1868
AIDS defining disease (yes vs. no)	− 0.0775	0.3714	0.8346
−168/PKR genotype (CT vs. CC)	0.8017	0.3860	**0.0378**
Age, years	− 0.0301	0.0275	0.2732
Duration of HIV infection	− 0.0040	0.0346	0.9091
Time	− 0.0077	0.0029	**0.0073**
Time: −168/PKR genotype (CT vs. CC)	0.0013	0.0035	0.7144

The term “time: −168/PKR genotype” indicates the parameter for the interaction term, allowing to describe the progression of CD8 for each −168/PKR allelic variant.

*WT/WT* wild type, *anti-HCV* hepatitis C virus antibodies, *AIDS* acquired immune deficiency syndrome, *PKR* protein kinase R.

Significant coefficients are in bold.

#### HIV-load dynamic and −168/PKR allelic variants

Also for HIV-load, two subgroups resulted as the optimal classification to capture biomarker dynamic over time: 14 participants (15.22%) were assigned to class 1 and 78 (84.78%) to class 2 (see Table S1).

Average posterior probabilities of falling into the class in which the subjects were classified are equal to 0.9006 and 0.9711, thus indicating an unequivocal classification.

By implementing a two latent classes model, we found that transmission through a sexual contact (p = 0.0374), coinfection with HCV (p = 0.0020) and AIDS diagnosis (p = 0.0009) were positively associated with HIV load (Table 4).

**Table 4:** Estimated latent class mixed model for HIV load (Log10 transformed) with two latent classes.

Parameter	Estimate	SE	p-value
Intercept class1 (not estimated)	–		
Intercept class2	− 1.0366	0.4831	0.0319
Sex (male vs. female)	0.0315	0.1760	0.8581
CCR5-delta32 polymorphism (*WT/WT* vs. *WT/delta32*)	0.1614	0.1698	0.3419
Risk factor for HIV infection (sexual vs. IVDU)	0.4575	0.2198	**0.0374**
Anti-HCV (pos vs. neg)	0.5747	0.1859	**0.0020**
AIDS defining disease (yes vs. no)	0.4785	0.1434	**0.0009**
−168/PKR genotype (CT vs. CC)-class1	1.0518	0.4442	**0.0179**
−168/PKR genotype (CT vs. CC)-class2	0.1731	0.2001	0.3869
Age, years	− 0.0146	0.0125	0.2442
Duration of HIV infection	− 0.0094	0.0134	0.4829
Time-class1	− 0.0281	0.0150	0.0610
Time-class2	− 0.0119	0.0016	** < 0.0001**
Time: −168/PKR genotype (CT vs. CC)-class1	0.0003	0.0150	0.9864
Time: −168/PKR genotype (CT vs. CC)-class2	− 0.0040	0.0019	**0.0365**

The term “time: −168/PKR genotype” indicates the parameter for the interaction term, i.e., it describes the progression of HIV load separately for each allelic variant and for each latent class (class1 or class2).

*WT/WT* wild type, *anti-HCV* hepatitis C virus antibodies, *AIDS* acquired immune deficiency syndrome, *PKR* protein kinase R.

Significant coefficients are in bold.

Although HIV-load decreased over time, a more marked decrease of HIV load was observed in heterozygous CT subjects than in homozygous CC subjects assigned to class 2 (p = 0.0365). Conversely, participants in class 1, exhibiting the CT variant, had on average, a higher HIV load than those with CC variant in the same class (p = 0.0179).

Class-specific model predicted curves for CD4, CD8 and HIV load highlighting the effect of PKR genotype in shaping biomarkers’ dynamics are reported in Fig. 1.

## Discussion

In biomedical research a main challenge is given by the search for interactions among factors that may affect final phenotype, uncovering hidden dependence structures in presence of heterogeneity. This is particularly true in HIV infections, where heterogeneity in disease outcome, due to differences in cohort characteristics, represents a major barrier to a more comprehensive understanding of the disease progression.

In this context, genome-wide association studies have been carried out to decipher host factors involved in the natural protection against HIV disease progression and several candidate genes have been shown to play a role in the disease phenotype^6^.

Previous results^16^ shown that heterozygosity at −168/PKR confers an advantage for spontaneous elimination of the virus during acute hepatitis C infection. Considering that HIV and HCV share similar mechanisms of PKR inhibition, we investigated the role of PKR allelic variants in the position −168 of the promoter region of PKR on the dynamic of immune-virological markers recognized to be associated with the progression of HIV disease.

HIV longitudinal data represent a major hurdle in observational studies, since are collected at non-constant time intervals, with different number of repetitions per patients and in presence of missing values. To overcome these problems and retain a high flexibility, we have implemented latent class mixed models (LCMMs) as an appealing modelling strategy to analyse even non-normally distributed longitudinal outcomes allowing to capture longitudinal dynamics. Within this framework, heterogeneity which is not fully explained by patients’ observed characteristics, is described by unobserved latent process. Thus, different subjects profiles can be drawn directly from the data. In particular we identified two longitudinal profiles of CD4 cell count.

Overall, we found that route of transmission (sexual exposure), HCV coinfection, diagnosis of AIDS and older age were negatively associated with CD4 cells count. Additionally, the heterozygous CT genotype in the promoter region of PKR emerged as a potential genetic factor associated with dynamic of CD4 and in particular a significant increase of CD4 cells count was observed in study participants with heterozygosity CT belonging to class 2 subgroup of HIV-carriers, but not in those assigned to class 1 subgroup.

Among the clinical and demographic variables analysed, sexual exposure, HCV coinfection and AIDS defining disease, were positively associated with HIV load overtime. It is worth noting that the role of PKR allelic variants on HIV load dynamic emerged only when considering a two latent classes model. Although HIV-load decreased over time, a more marked decrease of HIV load was observed in heterozygous CT subjects than in homozygous CC in the class 2 subgroup. Conversely, HIV-carriers with the CT variant in class 1 had on average higher HIV load than those with CC variant belonging to the same class.

In interpreting this apparently debatable result related to the class-specific effect of CT variant on HIV load we should consider that CC/CT allelic variants effect may differ in different estimated subgroups. To better explain this finding, additional covariates (e.g., genetic, demographic or clinical characteristics) should be evaluated to explain class membership and the corresponding different behavior over time within the latent classes. Furthermore, the sample size plays also a crucial role in the respective class identified by the model. In particular, the class 1 showing higher average HIV load in the presence of CT allelic variants included 14 study participants, of which, 10 were characterized by CT variant. Notably, in the class 2, a larger group of 78 study participants (of which, 53 were characterized by CT variant) were captured and several of these subjects had a better HIV load outcome according to CT allelic variant.

Concerning the association of biomarkers dynamics (CD4 cells and HIV load) with the route of transmission, several reports showed that to have acquired HIV infection through the sexual contact, was associated with a markedly better immune and virological response than to be infected with other transmission routes^18,19^. In particular, intra venous drug users (IVDUs) are reported to have unsatisfactory immune and virological outcomes, probably as a consequence of lower adherence to ART^20,21^. In our cohort, we found that sexual exposure is related to lower CD4 count and higher HIV load over time. It is possible that several sexually exposed individuals concomitantly used illicit substances or had high alcohol intake that ultimately may reduce adherence to treatment. However, in this study we did not precisely evaluate the possible use of illicit substances or alcohol intake that could influence the adherence and/or modify the kinetic of the drugs as part of the ART regimen.

The possible contribution of an HCV infection on the dynamic of biomarkers associated with HIV disease progression is not clearly defined. In the EuroSIDA cohort, no association between virological and immunological responses to highly active antiretroviral therapy (HAART) and HCV serostatus was found^22^; however, HCV coinfection has been associated with a higher rate of drug discontinuation in the same cohort^23^. A meta-analysis of eight trials showed that the mean increase in the CD4 cells count in HIV/HCV coinfected study participants was 33 cells/mm3 less than that for HIV-infected study participants without HCV infection^24^ and these results have been confirmed in one other meta-analysis in which also an increase in AIDS events was seen in coinfected subjects^25^.

Concerning the finding of a worse recovery of CD4 cells in older PLWH, it could be the consequence of immune senescence and/or CD4 exhaustion as part of long-term immune dysregulation^26,27^. This finding has been observed also in elderly subjects without HIV infection^28^. Otherwise, comorbidities and polypharmacy, could have contributed to a worse immune as well as virologic outcome in older PLWH^29^.

Finally, with reference to CD8 cells, instead, no latent subgroups were identified by our modelling approach: the hypothesis of a unique longitudinal profile was supported by the data and on average higher CD8 cells were observed for heterozygous CT subjects.

Altogether, these findings emphasize the importance of going beyond traditional adjustment for covariates and moving towards more effective alternative solutions within latent variable modelling framework, whenever the goal is accounting and modeling data heterogeneity.

We remark that in this study only Caucasian population strata was included: since genetic differences may exist among ethnic groups, our findings need to be validated in larger trials including different ethnicity. This allows to define strategies for new therapies and identify new biomarkers for HIV progression. The underlying mechanism by which the CT allele variant determines a more favorable virologic and immunologic outcome needs to be investigated by a mechanistic approach.

In conclusion, we confirmed the effect of clinical variables known to be associated with a worse immunologic and virologic outcome. By applying a latent classes mixed model, we were able to show an association of heterozygosity CT within the promoter region of PKR, relative to the transcription initiation site, with the dynamic change of CD4, CD8 and HIV load in study participants.

Our findings showed a more favorable immunologic and virologic outcome in study participants with −168/PKR CT variant respect to those with CC variant, especially when considering specific latent classes. Future research should be carried out to deeply characterize the emerged latent classes. In particular, other genes which have been shown to affect disease progression in addition to demographic and clinical covariates, could be explored to refine class membership and factors associated with biomarkers dynamic within subgroups.

## Materials and methods

### Study design and data collection

This is a retrospective study conducted on Caucasian PLWH attending the Infectious Diseases Department of the San Raffaele Scientific Institute as outpatients. Longitudinal data were collected from 2005 to 2018 or at last visit available. The following data were collected at baseline and during the period of observation: age, sex, route of transmission of HIV-1 infection, clinical category at diagnosis according to the Centers for Disease Control and Prevention (CDC) case definition, years of HIV infection, years of antiretroviral therapy (ART), HIV load, CD4 and CD8 lymphocyte count. Hepatitis B (HBV) and hepatitis C (HCV) coinfection was assessed by HBV surface antigen (HBsAg) and HCV antibody (anti-HCV) positivity, respectively. Data were collected as part of routine clinical care and recorded in the database of the Division of Infectious Diseases of the San Raffaele Hospital (CSLHIV Cohort). At their first visit in our center, all study participants signed an informed consent (also approved by the ethics committee of the San Raffaele Scientific Institute) to use their data for research purposes and to be included in the database of our department (CSLHIV-Cohort). Recorded data are anonymized and managed according to the Good Clinical Practice Guidelines published by the World Medical Association Declaration of Helsinki.

All methods were carried out in accordance with relevant guidelines and regulations.

We decided to consider in the analysis the subjects with at least two determinations of CD4, CD8 cells and HIV viral load (VL) in whom was characterized the allelic variant at position −168 of the PKR promoter region, as well as the CCR5-delta32 genotype. These gene variants were explored in year 2006–2007 in the context of HIV/hepatitis C virus studies^30^. All experimental protocols were approved by San Raffaele Scientific Institute local Institutional Review Board.

The molecular approach to detect these gene variants and data on their distribution in healthy subjects and PLWH are detailed in supplementary information (Fig. S1 and Table S2). Concerning the allelic variants at position −168 of the PKR promoter region, three variants has been detected in healthy individuals, CC CT and TT, with different distribution (see supplementary information). Since only one subject living with HIV harbored the TT allelic variant, we excluded this subject from the analysis.

At baseline evaluation all study participants were on ART or commenced ART, HIV-RNA load was measured by a different biomolecular assay as part of routine laboratory tests from year 2005 to 2008, while from year 2009 to year 2018 the biomolecular assay was maintained the same. However, a positive result was invariably assessed as an HIV-RNA level ≥ 50 copies/mL. Virological failure was assessed according to international guideline (https://www.eacsociety.org/media/final2021eacsguidelinesv11.0_oct2021.pdf).

### Statistical analysis

In Table 1 descriptive characteristics at baseline are reported for both, quantitative variables (median and interquartile range, IQR) and qualitative variables (frequencies and percentages).

Latent class mixed models (LCMMs^17^) have been applied to model longitudinally measured biomarkers of HIV progression with the aim of capturing heterogeneity in their dynamics while possibly uncovering group-specific profiles of response.

The specification of LCMMs requires the definition of a structural latent model, which is a linear mixed model without measurement errors, where the quantity of interest (a latent process) is modelled according to time and covariates, along with a measurement model describing the (non) linear relationship between the longitudinal outcome and the latent process. By structuring the model in this way, LCMMs result to be highly flexible in handling complex possibly unknown functional relationships. A multinomial logistic regression is also implemented to model latent class-membership based on covariates of interest. Random intercept and random slope models were specified. To better capture biomarkers dynamic, nonlinear link functions were defined (splines transformation with 5 equidistant knots). LCMMs with from 1 to 3 latent classes were estimated.

Several statistical criteria have been applied to select the model with the best fit and best clustering of the data, i.e., with the optimal number of latent classes.

In particular, we focus on the Integrated Classification Likelihood (ICL) criterion adopted when both model fitting and identification of natural groupings in the data are relevant for the analysis^31,32^. ICL criterion is defined as a combination of both the Bayesian Information Criterion (BIC) criterion and the posterior class membership and lower ICL values are associated with better model fit. To choose the final model we also account for the classes size. Hence, whenever the ICL criterion suggested a model with G classes where the number of observations in one or more classes was really low, the strategy was to choose as final model the immediately preceding model with (G-1) classes. The rationale for this choice was to obtain a model characterized by latent classes with enough observations to perform further descriptive or inferential analyses.

With reference to model specification, CD4 and CD8 cells count (square root transformed) along with HIV-1 viral load (Log10 transformed) represented the outcome variables of interest explained by the following covariates: distance in months between study entry and biomarker assessment date (referred to in what follows as “time”), age, sex duration of HIV infection, HCV co-infection, CCR5-delta32 polymorphism (WT/delta-32 vs. WT/WT) and route of HIV transmission (sexual contact or intra venous drug use, IVDU), PKR allelic variants (CC or CT).

All these covariates were entered in the models as fixed effects. Moreover, to allow for different outcome progression over time depending on −168/PKR allelic variants, an interaction term between time and −168/PKR allelic variant was also specified. When considering at least two latent classes, class-specific terms for time, PKR genotype along with their interaction were entered in the model. All of the analyses were performed using R statistical software (version 4.0.5). The significance level was set at 0.05. The R package lcmm^17^ was used to estimate the latent class mixed models.

## Supplementary Information

Supplementary Information.

## Data Availability

Data that support the findings of this study are available from the corresponding author on reasonable request.
