# Supplementary material for: Modelling the impact of protein-kinase R allelic variant on HIV biomarkers trajectories by means of latent class mixed models
Source: Sci Rep. 2022 Nov 3;12:18575. doi: 10.1038/s41598-022-23289-4 (PMC9633692; doi:10.1038/s41598-022-23289-4)

Supplementary information to “Modelling the impact of protein-kinase R allelic variant on HIV biomarkers trajectories by means of Latent Class Mixed Models”

Chiara Brombin^1,2*^, Sabrina Bagaglio^3*^, Federica Cugnata^1,2^, Antonella Castagna^2,3^, Caterina Uberti-Foppa^2,3^, Stefania Salpietro^3^, Clelia Di Serio^1,2,4^, Giulia Morsica^3^

^1^University Centre for Statistics in the Biomedical Sciences (CUSSB), Milano, Italy

^2^Vita-Salute San Raffaele University, Milano, Italy
^3^Department of Infectious Diseases, IRCCS San Raffaele Scientific Institute, Milan, Italy

^4^Biomedical Faculty, Università della Svizzera Italiana, 6900 Lugano, Switzerland

*these authors have contributed equally to this work

**Detection of PKR and CCR5 delta-32 variants**

Peripheral blood mononuclear cells (PBMCs) were isolated by means of Ficoll-Hypaque density gradient centrifugation, and genomic DNA was extracted from pellets containing 10^6^ PBMCs using a commercially available kit (QIAamp DNA Mini Kit, QIAGEN SpA, Milan, Italy) according to the manufacturer’s instructions.

A 187 bp fragment corresponding to the partial region of the PKR promoter (466-645 of HSU51035) was amplified using a nested PCR (outer: forward Pro0 447-465, 5’-TCCTGGCCGTGCAGGGGCA-3’; reverse Pro3 690-673, 5’-GCCGGCCGGAGACCCGCG-3’;inner: forward Pro1 467-484, 5’-ACGTGGGTGCCAAGCCCG-3’; reverse Pro2 671-654, 5’-CTTCGGGAGAGCTGGTTC-3’. The PCR products were electrophoresed on a 1.5% agarose gel stained with DNA-star (Lonza) dye and visualized under UV light.

Allelic polymorphism (CC, CT, TT) at position -168 from the transcription start of the PKR gene was investigated by means of direct sequencing of the PCR products by using Pro2 inner reverse and electropherogram analysis of complementary strain. All data were confirmed in at least two different experiments. Genotyping of the -168 allelic variant by polymerase chain reaction (PCR) and DNA sequencing are demonstrated in Figure S1.

For genotyping the *CCR5* [the wild (*WT/WT*) and mutant (*WT/delta32*)] PCR method was applied, as described by Wu et al. (8). The PCR products were electrophoresed on a 3% agarose gel stained with DNA-star (Lonza) dye and visualized under UV light.

The study was conducted in 136 healthy individuals (HI) and 93 people living with HIV (PLWH) to assess the proportion of PKR genotypes in Caucasian HI and PLWH.

Results are summarized in Table S2.

The Hardy-Weinberg equilibrium was respected (chi-square=0.09; p=0.7642) in the control group of HI.

**Table S1**: Criteria used for model selection: Integrated Classification Likelihood (ICL) criterion along with latent class proportion (%, class membership) for the estimated models for each outcomes.

| **Number of classes** | **ICL** | **%class1** | **%class2** | **%class3** |
| --- | --- | --- | --- | --- |
| **CD4-1 class** | 15106.55 | 100 |  |  |
| **CD4-2 classes** | 14952.33 | 26.086957 | 73.91304 |  |
| **CD4-3 classes** | 14942.63 | 5.434783 | 83.69565 | 10.86957 |
|  |  |  |  |  |
| **VL-1 class** | 6125.114 | 100 |  |  |
| **VL-2 classes** | 5945.351 | 15.217391 | 84.78261 |  |
| **VL-3 classes** | 5957.319 | 7.608696 | 61.95652 | 30.43478 |
|  |  |  |  |  |
| **CD8-1 class** | 14770.02 | 100 |  |  |
| **CD8-2 classes** | 14588.51 | 94.56522 | 5.434783 |  |
| **CD8-3 classes** | 14609.23 | 25 | 69.565217 | 5.434783 |

**Table S2**: Distribution of allelic variants CC/CT/TT at position -168 of the promoter region of protein kinase R (-168/PKR) in healthy individuals (HI) and PLWH.

|  | **HI**  **(n=136)** | **PLWH**  **(n=93)** |
| --- | --- | --- |
| Age, years median [IQR] | NA | 43 [40-47] |
| Gender Male (%) | 86 (63.2) | 71 (76.3) |
| CC (%) | 48 (35.3) | 29 (31.2) |
| CT (%) | 67 (49.3) | 63(67.7) |
| TT (%) | 21 (15.4) | 1 (1.1) |

**Figure S1**. Identification of allelic variants (CC/CT/TT) at position -168 of the PKR-promoter.

(A) Polymerase chain reaction products were run on 1.5% agarose gel and DNA bands stained by

ethidium bromide. Under these conditions a 187-bp band was detected (sample S1-S13). Lane M shows the molecular weight marker. (B) Electropherogram analysis. The PCR fragments were sequenced by using reverse primer, therefore, in the electropherogram, complementary nucleotides are shown. Homozygous genotype CC was read on electropherogram as a single black peak. Black color identifies G nucleotide which is complementary to C. Homozygous genotype TT was read on electropherogram as a single green peak. Green color identifies A nucleotide, which is complementary to T. The heterozygous genotype CT is detected as the presence of two peaks of similar height and amplitude, at the position -168 of PKR promoter (green peak, corresponding to A plus black peak corresponding to G): the nucleotide at position -168, is indicated as N in the sequence.


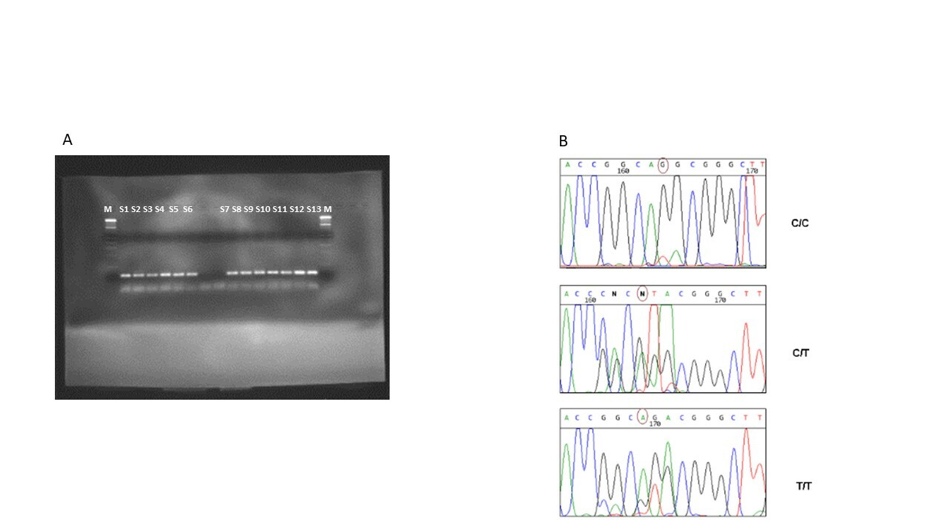

Supplement: Supplementary file 1 — Supplementary Information. [file 41598_2022_23289_MOESM1_ESM.docx]
